# Fructose Production by Inulinase Covalently Immobilized on Sepabeads in Batch and Fluidized Bed Bioreactor

**DOI:** 10.3390/ijms11031180

**Published:** 2010-03-19

**Authors:** Emanuele Ricca, Vincenza Calabrò, Stefano Curcio, Alessandra Basso, Lucia Gardossi, Gabriele Iorio

**Affiliations:** 1 Department of Engineering Modeling, University of Calabria, I-87036 Arcavacata di Rende, CS, Italy; E-Mails: ericca@unical.it (E.R.) calabrov@unical.it (V.C.); gabriele.iorio@unical.it (G.I.); 2 SPRIN S.p.A., c/o BIC, Via Flavia 23/A, 34100 Trieste, Italy; E-Mail: basso@sprintechnologies.com; 3 Laboratory of Applied and Computational Biocatalysis, Dipartimento di Scienze Farmaceutiche, Università degli Studi, P.le Europa 1, 34127 Trieste, Italy; E-Mail: gardossi@units.it

**Keywords:** enzyme reaction, inulin hydrolysis, fluidized bed reactor, fructose

## Abstract

The present work is an experimental study of the performance of a recently designed immobilized enzyme: inulinase from *Aspergillus* sp. covalently immobilized on Sepabeads. The aim of the work is to test the new biocatalyst in conditions of industrial interest and to assess the feasibility of the process in a fluidized bed bioreactor (FBBR). The catalyst was first tested in a batch reactor at standard conditions and in various sets of conditions of interest for the process. Once the response of the catalyst to different operating conditions was tested and the operational stability assessed, one of the sets of conditions tested in batch was chosen for tests in FBBR. Prior to reaction tests, preliminary fluidization tests were realized in order to define an operating range of admissible flow rates. As a result, the FBR was run at different feed flow rates in a closed cycle configuration and its performance was compared to that of the batch system. The FBBR proved to be performing and suitable for scale up to large fructose production.

## 1. Introduction

Fructose production from inulin enzymatic hydrolysis is a promising process, which has gained remarkable attention in recent years because it brings higher fructose concentrations than the traditional process based on glucose isomerization. In fact, the latter is a reversible reaction and fructose equilibrium concentration cannot be higher than 50% of total sugars; the techniques used to push fructose concentrations above that limit are based on chromatography and are very costly [1]. Inulin hydrolysis, instead, is an irreversible reaction and it can yield fructose concentrations of up to 95% [2] in one step, without further separations.

A recent work by Ricca *et al.* [3] reviews the main advances of research in this field. It can be noticed that aspects related to the substrate (inulin) and the catalyst (inulinase) are very deeply dealt with in the Literature. Many vegetable sources for inulin have been reported [4–6] as well as extraction techniques [7–9]; as far as the enzyme is concerned, there are many microbial sources [10] and its properties in terms of mode of action, activity, stability and kinetics are also known [3]. On the contrary, application aspects, such as the use of immobilized enzyme in continuous reactors, need improvement and a larger number of proposals in order to find the best solution for industrial purposes.

It is worth noticing that only few papers have appeared so far on continuous processes with immobilized inulinase and in most of them packed bed reactors (PBRs) were used, with few exceptions such as the work by Diaz *et al.* [11] who employed a shell-tube membrane reactor. Among the most recent works on PBRs, remarkable results were obtained by Gupta *et al.* [12] who used inulinase immobilized on DEAE-cellulose, Nakamura *et al.* [13] who used Amino-cellolufine as the support, Wenling *et al.* [9] who used macroporous ionic polystyrene beads and by Gill *et al.* [14] who tested different supports: chitin, QAE-Sephadex and ConA linked-amino activated silica beads.

A new type of support for enzyme immobilization is drawing great attention in recent years: Sepabeads®, a class of methacrylic polymers very suitable for enzymes immobilization for industrial purposes [15]. They have already been employed in a number of applications: Ghazi *et al.* [16] successfully used them for immobilization of fructosyltransferase for the synthesis of fructo-oligosaccharides from simple sugars, Alonso *et al.* [17] for immobilization of glutaryl acylase and Basso *et al.* [18] for immobilization of CalB (*Candida antarctica* B-lipase) and PGA (*Penicillin* G Acylase).

Previously, Basso *et al.* [19] reported a rational immobilization of inulinase on Sepabeads® based on homology modeling, docking and molecular dynamics, GRID analysis. In the present work, the new preparation of immobilized enzyme is tested in conditions of industrial interest and a reactor configuration innovative in the field of fructose production from inulin is proposed and tested. Experiments were run in batch and continuous reactors. Batch runs were carried out in order to test the enzyme performance in different conditions of temperature, substrate concentration and amount of enzyme used; reuse tests were also performed to check the operating stability of the catalyst.

Regarding the continuous process, this work reports the first attempt to employ a biocatalyst immobilized on Sepabeads in a fluid bed reactor. Some preliminary fluidization tests were necessary to establish the suitability of the supporting material to fluidization and, afterwards, proper reaction tests were run at different flow rates fed to the reactor. Comparison of performances among tests at different conditions and between batch and FBR are also reported.

## 2. Materials and Methods

### 2.1. Materials

The substrate used was inulin from chicory (cod. I2255, Sigma Aldrich, Italy). The characterization of the substrate in terms of degree of polymerization (DP) was obtained by means of inulin complete acidic hydrolysis, according to a procedure described in a previous work [20] and a DP of 28 was found, corresponding to 4700 Da molecular weight.

The enzyme was a commercial liquid mixture (ρ = 1.13 g/mL) of exo- and endo-inulinases (Fructozyme L™) kindly provided by Novozymes A/S (Denmark); the declared activity was 2000 U/g. Immobilization carriers were Sepabeads^®^, methacrylic polymers produced and commercialized by Resindion S.r.l. (Mitsubishi Chem. Corp, Milano, Italy), supplied with a water content of about 70% w/w. Sepabeads^®^ with oxirane groups were used in the present work. The immobilization procedure was implemented with SPRIN, Trieste (www.sprintechnologies.com).

All the chemicals used for acetate buffers preparation with distilled water (acetic acid, sodium acetate trihydrate) were reagent grade.

### 2.2. Enzyme Immobilization

#### Immobilization procedure

The immobilization was carried out at 25 °C and 40 rpm in a blood rotator in KH_2_PO_4_/K_2_HPO_4_ Kpi buffer (1.25M, pH 8.0), as described previously [20]. 77 U of Fructozyme L per gram of wet polymer were used with a support/buffer ratio of 1/4 w/v. The immobilization proceeded for 19h, under constant stirring, then for 21h without stirring. The solution was filtered and the liquid phase was recovered for protein determination. The immobilized protein was washed with Kpi buffer (0.02M, pH 8.0, ratio support/buffer 1/4 w/v). Adsorbed proteins were desorbed by adding 0.5M NaCl in Kpi buffer (0.02M pH 8.00, ratio support/buffer 1/4 w/v) and then by stirring for 45 min. The preparations were finally rinsed with Kpi buffer (0.02M, pH 8.0, ratio support/buffer 1/4 w/v) and the activity was checked. The immobilized enzymes were stored at 4 °C.

#### Inulinase activity

The assay is based on the hydrolysis of inulin to fructose. A solution of inulin in acetate buffer (0.1M, pH 5.0) with a concentration of 10 g/L was prepared and maintained at 50 °C. The immobilized enzyme was added to the solution of inulin and the suspension was stirred for a variable time from 15 to 30 minutes. To stop the reaction, the suspension containing the immobilized enzyme was filtered.

The solution was refrigerated to room temperature and analysed by HPLC. The activity was calculated on the basis of a calibration curve and defined as:

$$\text{Activity}=\frac{\mu \text{mol}\text{Fructose}}{\text{min}\times g\text{dry}}$$

#### Protein Determination

The protein content of Fructozyme L (25.17 mg/mL) was determined by using bicinchoninic acid kit (SIGMA)-Pierce method, using BSA as protein standard and UV measurements performed with a Lambda 20 UV/Vis Perkin–Elmer spectrophotometer.

The percentage of bound enzyme was determined as the difference between the concentration of the native protein before immobilization and in the filtrates after immobilization. The protein amount was determined by Pierce method, using inulinase solution for calibration curve [21].

#### Determination of Water Content

The water content of each preparation was evaluated by drying the samples at 110 °C for 6 h on aluminium dishes and by determining the difference in weight between the wet and the dried sample.

### 2.3. Batch Experiments

Batch tests were run in a system (Applikon, The Netherlands) consisting of: a 1.5 L glass vessel, a six-blade impeller driven by an electric motor (Stirrer Motor Assembly P100) controlled by a stirrer controller (P100, ADI 1032), pH and temperature sensors, a jacket for temperature control by means of a Bio Controller (ADI 1030).

The reaction volume was 500 mL, pH was kept to 5.0 by means of an acetate buffer and its values were constantly measured and all other variables were changed as follows: S_0_ = 10 and 40 g/L, T = 40 °C and 50 °C, E_0_ =1 and 4 g_cat_.

The reuse cycle of the enzyme was performed after 28 h of operation and after filtering the biocatalyst with distilled water.

Reaction was quenched by separating the enzyme from the reacting mixture by means of a 90 μm filter and samples were immediately analyzed.

### 2.4. FBBR Continuous Operation

**Figure 1.**
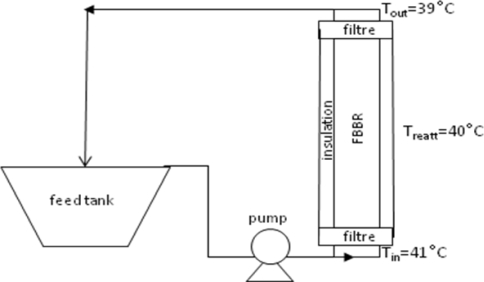
Schematic representation of experimental apparatus.

Continuous inulin hydrolysis was performed in a fluidized bed bio-reactor (FBBR) consisting of a Plexiglass® tube of 50 mL (1.6 cm diameter, 25 cm length). At both ends of the reactor, 90 μm filters were fitted in order to avoid possible elutriation of immobilized enzyme from the reaction environment. The reactor was charged with E_0_ = 1 g_cat_ of immobilized enzyme and fed with an inulin solution at pH 5.0 and S_0_ = 10 g/L. The feed flow rate ranged between 1.6 and 4 l/h and the reactor was operated in a closed cycle configuration. The temperature was kept at 40 °C by pre-heating the feed stream and insulating the bioreactor. Temperature values were measured at both ends of the bioreactor in order to verify the existence of isothermal conditions. A schematic representation of the FBBR device is given in Figure 1.

### 2.5. Analysis of Reaction Products

Sugar analysis was carried out by high performance liquid chromatography at room temperature. The mobile phase was aqueous H_3_PO_4_ 0.1% v/v, fed at a flow rate of 0.5 mL/min. The column was a Supelcogel C-610H. The detection relied on refractive index (RI 930 Jasco).

## 3. Results and Discussion

### 3.1. Batch Tests

**Figure 2.**
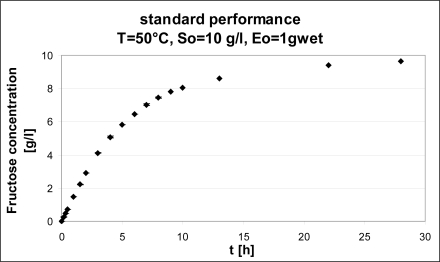
Standard performance of immobilized enzyme on long time scale (t = 28 h).

The first test was performed at the conditions of the activity assay, but on a long time scale, in order to achieve a basis for comparison at different conditions (Figure 2).

At standard conditions, the enzyme is able to push the reaction to almost completeness in 28 hours, with production of 9.6 g/L of fructose from 10 g/L of inulin (corresponding to 88% conversion); test reproducibility is remarkable (error bars based on standard deviation are reported in Figure 2, and are always smaller than 1% of the mean value).

A subsequent test was performed in which the catalyst loading was changed from 1 to 4 g_cat_, to observe the effect of a larger amount of enzyme. It was found that the reaction rate was, of course, higher, but less than proportional with respect to the increase in catalyst quantity. In order to have a reliable comparison, the normalized reaction rate (*i.e.,* referred to the weight unit of catalyst) was calculated. The results are reported in Table 1.

The specific activity data show that when 4g_cat_ are used the performance is lower than the case of 1g_cat_, and this is because when the enzyme loading is too high some of the catalyst is not exploited, lowering the process productivity.

**Figure 3.**
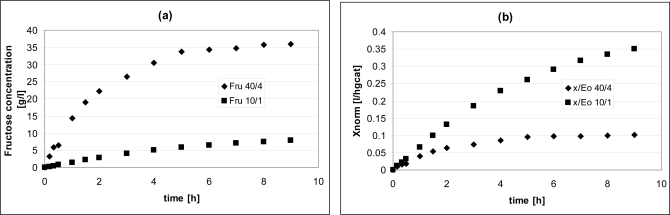
Performance comparison at S_0_/E_0_ = 10/1 (rhombi) and S_0_/E_0_ = 40/4 (squares) in terms of fructose concentration (a) and normalized inulin conversions (b).

By the last observation, it was decided to use E_0_ = 4 g_cat_ and S_0_ = 40g/L in order to have a higher amount of enzyme, but the same S_0_/E_0_ ratio as in the standard test (Figure 3).

In this case, the absolute difference in terms of concentration of fructose produced between the two instances is even larger (Figure 3a), but if results are reported in terms of normalized inulin conversion $\left(x_{\text{norm}}=\frac{S_{0}-S}{S_{0}}\frac{1}{E_{0}}\right)$, as a measure of the real extent of reaction per catalyst unit, then the situation is reversed (Figure 3b). The value of specific normalized activity (*i.e.*, initial slope of data in Figure 3b) for the case S_0_/E_0_ 10/1 is 0.054 1/(g_cat_h), while for 40/4 it is 0.067. This proves that the ratio S_0_/E_0_ is not a significant variable for the process, because individual changes of E_0_ and S_0_, with S_0_/E_0_ kept constant, determine different performance.

A reuse test has also been performed at 50 °C in order to evaluate the operational stability of the catalyst. The enzyme was firstly used for 28 h in standard conditions (see Figure 2) and then its activity was tested as initial reaction rate: after 28 h the enzyme retained 85% of activity. Eventually, all other standard conditions unchanged, temperature was switched to 40 °C. The effect was that the catalyst could operate at a temperature at which thermal deactivation is less likely to occur without a great decrease of the reaction rate; in fact, the initial rate of reaction in these conditions is 1.30 (g/(l h)) as compared to 1.46 at 50 °C, *i.e,.* 84% of the standard performance.

### 3.2. FBBR Tests

**Figure 4.**
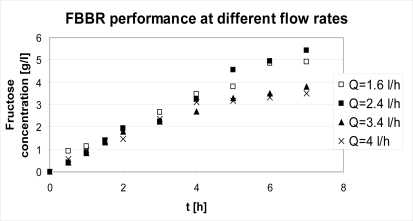
FBBR performance at different flow conditions; T = 40 °C, S_0_ = 10 g/L, E_0_ =1 g_cat_.

The first aspect to investigate in a continuous process perspective was the suitability of Sepabeads® to fluidization. For this reason, preliminary test of fluidization were performed and it was found that for the system under study a certain range of flow rates was feasible, relying on the granulometric dispersion of the material (150–300 microns). Based on those preliminary tests, it was decided to run the FBR under four different flow conditions: 1.6 l/h, 2.4 l/h, 3.4 l/h, 4 l/h. The experimental results found are reported in Figure 4.

Even though at higher flow rates a better mixing is expected with a subsequent higher efficiency of the catalyst due to low mass transport resistances, the best performances are displayed at low flow rates. Since the bioreactor is operated in closed cycle configuration, the mean residence time within the reactor is independent of flow rate. This suggests that better performance at low flow rates is due to the absence of mechanical stresses on the catalyst that instead occur at higher flow rates. This is also confirmed by the observation that the differences among the four cases are not relevant in the first stages of the reaction where the experimental points are almost overlapping, while they become noticeable after four hours when the effect of stresses starts emerging.

**Figure 5.**
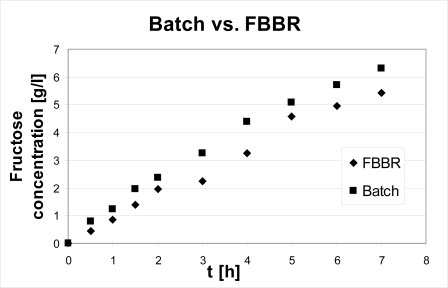
Continuous *versus* discontinuous reactor

The best trend in terms of fructose concentration within the product stream is obtained at a flow rate of 2.4 l/h and it is compared to batch results. As a basis for comparison, the following conditions were chosen: S_0_ = 10g/L, E_0_ = 1g_cat_, T = 40 °C. The comparison between the discontinuous reactor (batch) and the continuous one (FBBR) relies on the fact that the latter is run in a closed cycle configuration, *i.e.,* the reactor is continuous, but the process as a whole is discontinuous, products being totally recycled to the feed tank; the volume of reacting mixture processed in this way does not depend on the flow rate to the reactor, but on the volume charged at the beginning of the operation: this volume was set to 500 mL in order to allow the comparison with a batch of the same volume. Results are shown in Figure 5.

The comparison shows that the performance of the FBR is not much lower than that of the batch. After seven hours, the latter produced 6.3 g/L of fructose, while the former produced 5.4. The reasons for these differences are: i) the mixing is better in the batch reactor and mass transport resistances external to the immobilized enzyme are responsible for a lower observed rate of reaction; ii) even if the comparison was made on the same volume base (500 mL of reacting mixture), the volume of the FBBR is only 50 mL with the remaining 450 mL due to the feed tank and, to a minimal extent, to connections between the reactor and the tank, thus only 50 mL of reacting mixture constantly encounter the catalyst. However, the former reason is quantitatively less relevant as the data shown in Figure 3 demonstrate that in the range considered the flow rate is not a decisive variable for the process; this means that in the fluid-dynamic conditions occurring within the reactor the external resistances are negligible as compared to reaction kinetics.

## 4. Conclusions

In the present work, an inulinase mixture (Fructozyme L™) immobilized on methacrylic carriers (Sepabeads®) was tested as a biocatalyst of industrial interest. The main topic of the paper was to assess the feasibility of a fluidized bed reactor with such immobilized enzymes as catalysts. This was done and the effect of changing the feed flow rate was observed; in particular, it was found that the effect of external mass transport was not so important in the conditions used, with reaction kinetics being controlling, in some cases limiting as it can be inferred by observation of results at 1.6 and 2.4 l/h, where the FBBR performance is not influenced by fluid-dynamic conditions. FBBR results were compared to batch experimental data showing a surprising performance of the former, only slightly lower than that of the batch system although in this case a major volume of reacting mixture was available at any time. Batch runs were performed at different conditions and with reuse of the catalyst suggesting an appropriate operating temperature of 40 °C for the biocatalyst.

The use of a fluidized system for fructose production from inulin not only is a new alternative to the reactor configurations so far proposed in the Literature, but, as outlined by other authors [22] about enzymatic transformations of natural raw materials, it could be of great help when treating unclarified solutions, whereas a packed bed might behave as a filter subjected to be clogged by the reacting mixture itself.

## Figures and Tables

**Table 1.** Enzyme performance at different loadings.

**Enzyme loading [g_cat_]**	**Initial velocity [g/Lh]**	**Specific initial velocity [g/g_cat_h]**
1	1.46	0.72
4	5.32	0.66
